# To Enhance Mucus Penetration and Lung Absorption of Drug by Inhalable Nanocrystals-In-Microparticles

**DOI:** 10.3390/pharmaceutics14030538

**Published:** 2022-02-28

**Authors:** Guiting Huang, Shuyuan Shuai, Weicheng Zhou, Yingchong Chen, Baode Shen, Pengfei Yue

**Affiliations:** Key Lab of Modern Preparation of TCM, Ministry of Education, Jiangxi University of Chinese Medicine, Nanchang 330004, China; jxiejx@outlook.com (G.H.); 1352341905@126.com (S.S.); yyang94@126.com (W.Z.); 20152016@jxutcm.edu.cn (Y.C.); 20211047@jxutcm.edu.cn (B.S.)

**Keywords:** nanocrystals-in-microparticles, mucus penetration, inhalation administration, TPGS

## Abstract

To effectively achieve the pulmonary delivery for curcumin (CN), novel inhalable mucus-penetrating nanocrystal-based microparticles (INMP) were designed. The D-Tocopherol acid polyethylene glycol 1000 succinate (TPGS) modified CN nanocrystals (CN-NS@TPGS) were prepared by high pressure homogenization and further converted into nanocrystal-based microparticles (CN-INMP@TPGS) using spray-drying. It was demonstrated that CN-NS@TPGS exhibited little interaction with the negatively charged mucin due to a strong electrostatic repulsion effect and PEG hydrophilic chain, and exhibited a much higher penetration ability across the mucus layer compared with poloxamer 407 modified CN-NS (CN-NS@P407) and tween 80 modified CN-NS (CN-NS@TW80). The aerodynamic results demonstrated that the CN-INMP with 10% TPGS acting as the stabilizer presented a high FPF value, indicating excellent deposition in the lung after inhalation administration. Additionally, in vivo bioavailability studies indicated that the AUC_(0-t)_ of CN-INMP@TPGS (2413.18 ± 432.41 µg/L h) were 1.497- and 3.32-fold larger compared with those of CN-INMP@TW80 (1612.35 ± 261.35 µg/L h) and CN-INMP@P407 (3.103 ± 196.81 µg/L h), respectively. These results indicated that the CN-INMP@TPGS were absorbed rapidly after pulmonary administration and resulted in increased systemic absorption. Therefore, the inhalable CN-INMP could significantly improve the bioavailability of CN after inhalation administration. The developed mucus-penetrating nanocrystals-in-microparticles might be regarded as a promising formulation strategy for the pulmonary administration of poorly soluble drugs.

## 1. Introduction

Pulmonary inhalation administration is not only an ideal way to locally treat lung diseases, such as chronic obstructive pulmonary disease, bronchitis and lung cancer [1,2,3], but also to achieve systemic absorption for other diseases owing to the great surface area (70–140 m^2^ in the adult human lung) of the lung and fast transport of active ingredients across the respiratory epithelium (relatively high blood flow up to 5000 mL/min) [4,5,6]. Unfortunately, pulmonary formulations were restricted by the physiological barrier of the lungs, such as the complex branching structure, mucociliary clearance and macrophages phagocytosis [7], as well as the particles size and solubility of the drugs [8]. Meanwhile, the aerodynamic requirements could prove to be a great challenge for effective lung deposition for inhalation formulation. The inhalable nanocrystals-in-microparticles (INMP), as an interesting dry powder-based inhalation formulation, has received increasingly more attention from researchers, which combines the advantages of nanocrystals (e.g., increased dissolution and enhanced absorption) [9,10] and microparticles (e.g., superior aerodynamic properties) [11,12]. Owing to its suitable aerodynamic characterization, the INMP exhibits more effective lung deposition in comparison with conventional nanoparticles, and can be reconstituted into drug nanocrystals when contacting mucus on the respiratory surface after deposition [13,14]. Moreover, the particles size and surface properties of nanocrystals can significantly influence their fate (absorption or clearance) in the lung. Once the drug is administered to the lungs, undissolved particles may be rapidly cleared by the mucociliary or engulfed by alveolar macrophages. The mucus plays an important role in mucociliary clearance, and the major functional components of mucus are the mucin glycoproteins presented at a concentration of 1–5% [15,16]. The mucin monomer can form a mucin fiber mesh with different mesh spacing diameters of 50–1800 nm in an aqueous medium, which depends on the cysteine bridge connection. The physicochemical properties of nanocrystals might affect the clearance of mucociliary. It was reported that poloxamer 188-coated nanocrystals with particle sizes of <500 nm could rapidly penetrate the respiratory mucus [17]. So, by suitable surfactant modification, the nanocrystals can escape the mucociliary clearance and reach the alveoli. As a water-soluble derivative of vitamin E and PEG1000, D-tocopherol acid polyethylene glycol 1000 succinate (TPGS) has been widely used as an effective stabilizer of drug nanocrystals [18]. Although TPGS may improve stabilization in vitro and the oral bioavailability of nanocrystals after administration, limited progress has been made on how to circumvent the interactions between nanocrystals and mucus, in order to improve retention in the lung after pulmonary administration.

**Figure 1:**
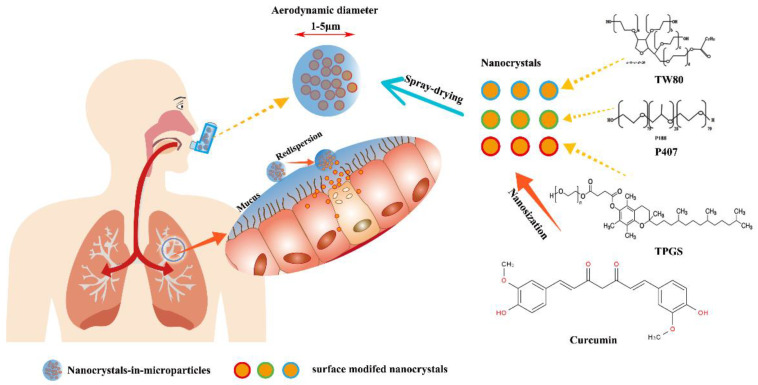
The schematic image on in inhalable nanocrystals-in-microparticles.

The curcumin (CN) was found to have strong anticancer, antioxidant, anti-inflammatory activities [19,20], but CN was a very poorly soluble drug, resulting in low bioavailability in vivo [21,22]. As illustrated in Figure 1, CN was used as the model drug in this study, and we designed a novel formulation strategy of inhalable mucus-penetrating nanocrystals-in-microparticles, which can easily escape adsorption of mucin glycoproteins and enhance the deposition and absorption of the drug in lung. The purpose of this study was as follows: (1) To prepare the curcumin nanocrystals-based suspensions (CN-NS) modified by P407, TW80 and TPGS using high pressure homogenization, respectively, and to evaluate physicochemical characterizations of different surface-modified CN-NS. (2) To investigate the interactions of different surface-modified CN-NS with mucus in order to evaluate the mucin adsorption and mucus-penetrating ability of CN-NS. (3) To convert the CN-NS into inhalable nanocrystals-in-microparticles (CN-INMP) using spray-drying, and to investigate the redispersibility, dissolution and aerosolization properties of the CN-INMP. (4) To study the drug retention in lung and the in vivo bioavailability of CN-INMP after pulmonary administration.

## 2. Materials and Methods

### 2.1. Materials

Curcumin (CN, purity > 98%) was purchased from Chengdu Herbpurify Biotechnology Co., Ltd. (Chengdu, China). The mucin was commercially purchased from Sigma-Aldrich (Shanghai, China). D-Tocopherol acid polyethylene glycol 1000 succinate (TPGS) was commercially purchased from Up-style Biotechnology Co., Ltd. (Xi’an, China). Both tween 80 (TW80) and poloxamer 407 (P407) were commercially purchased from Beijing Fengli Jingqiu Pharmaceutical Co., Ltd. (Beijing, China). Alexa Fluor-488-WGA was obtained from Thermo Fisher Scientific (Shanghai, China). Lactose (LA) were kindly obtained from Dingsheng Chemical Co., Ltd. (Nanchang, China). Beas-2B cell and A549 cell lines, cell counting kit-8 (CCK-8) were purchased from KeyGEN BioTECH Co., Ltd. (Nanjing, China).

### 2.2. Methods

#### 2.2.1. Preparation of CN-NS and CN-INMPs

The CN-NS were prepared according to the reported method with some modification [23]. Briefly, 1 g of coarse CN was dispersed in 100 mL 10% of TW80, P407 and TPGS solution (*w*/*w*, relative weight to CN), respectively. The CN nanocrystals suspensions (CN-NS) were then prepared using AH100D high-pressure homogenizer (ATS Engineering Inc., Shanghai, China) at 800 bar pressure for 40 cycles. Thereafter, the different concentrations of lactose (*w*/*w*) as matrix formers was added into the CN-NS, respectively. Spray-drying was performed using a Büchi spray dryer B-90 (Buchi Laboratoriums-Technik AG, Flawil, Switzerland). The gas inlet temperature and flow rate were set to 75 °C and 4 mL/min, respectively. The atomizing air flow was at 55 mmHg. The CN-INMP was deposited at the bottom of the collector. The samples were stored in a desiccator at room temperature for further evaluation.

#### 2.2.2. Particles Size and Zeta Potential Assay of CN-NS

The particles size of CN-NS was measured by a Mastersizer Micro Plus (Malvern Instruments Limited, Worcestershire, UK). The Zeta potential of the CN-NS was determined using the Zetasizer Nano-ZS (Malvern Instruments, Malvern, UK). All samples were measured in triplicate at 25 °C.

#### 2.2.3. Transmission Electron Microscopy (TEM)

Transmission electron microscopy (TEM) was used to observe the morphology of CN-NSs. The samples were dropped onto a copper grid (Beijing Zhongjingkeyi Technology Co., Ltd., Beijing, China) and dried in air. The copper grid loading CN-NS was observed using the JEM-1200EX (JEOL, Tokyo, Japan).

#### 2.2.4. In Vitro Interaction between CN-NS and Mucin

The mucin was dissolved in PBS (1 mg/mL) and stirred for 2 h. Then, 5 mL different CN-NS solution was reacted with a 1 mL mucin solution (2 mg/mL) at 37 °C for 4 h in a shaker (150 rpm). The size and zeta potential of the mucin-NS complex at a predetermined time point were measured using the Zetasizer Nano-ZS (Malvern Instruments, UK) [24].

#### 2.2.5. Mucus Diffusion Analysis

Furthermore, a 24-well transwell system with 0.4 μm pores (Corning Incorporated, Corning, NY, USA) was used to assess the mucus-penetrating ability of the CN-NS according to the previously reported method [25]. Briefly, 50 µL of mucus (10 mg/mL) suspensions was placed uniformly on the polycarbonate membrane of transwell. Then, 0.5 mL of the phosphate-buffered solution (pH 6.8) was placed into the acceptor compartment, and 60 µL of CN-NS/TPGS, CN-NS/TW80, and CN-NS/P407 was added to the donor chamber. The transwell plate was incubated in a shaker (100 rpm) at 37 °C for 2 h. Then, the transwell system was incubated at 37 °C in a shaker (50 rpm). Afterwards, 50 µL of the sample was collected from the acceptor chamber, and an equal volume of phosphate-buffered solution was instantly replenished. The fluorescence intensity of penetrated CN-NS was measured using a microplate reader. The apparent permeability coefficient (*P_app_*, cm/s) of CN-NS was evaluated according to the equation below:

$$P_{app}=\frac{d_{Q}}{d_{t}}*\frac{1}{A*C_{0}}$$

where, the *d_Q_*/*d_t_* represents the diffusing rate of different surface modified CN-NS.

A represents the area (cm^2^) of the membrane. *C*_0_ represents the initial CN-NS concentration in the donor compartment.

#### 2.2.6. D Visualization Evaluation of Particles Penetration Ability

To observe the penetration ability of CN-NS, the mucus suspension was firstly stained with Alexa 488-WGA (10 μg/mL) for 10 min in a shaker (150 rpm) at 37 °C. After that, 1 mL of stained mucin was deposited into a confocal dish, and placed on a shaker (50 rpm) in order to generate mucus layers with equal thickness. The CN-NS (100 μL) were carefully added dropwise onto the mucus layer and incubated for 30 min at 37 °C. The 3D images were captured every 20 μm along the Z-axis using a CLSM (Leica TCS SP8 X, Wetzlar, German).

#### 2.2.7. The Reconstitution Ability of CN-INMPs

The redispersibility of CN-INMP in simulated lung fluid was evaluated according to the previously reported method [26]. To do this, 100 mg of CN-INMP was placed into a tube, and then 10 mL of simulated lung fluid was added. The tubes were gently vibrated in a shaker (150 rpm) for 30 min at 37 °C. The particle size of suspensions were measured by a Mastersizer Micro Plus (Malvern Instruments Limited, Worcestershire, UK). Additionally, the redispersibility index (RDI) was obtained according to the previously reported method [12,26].

#### 2.2.8. In Vitro Drug Release of CN-INMPs

The in vitro release of CN from different CN-INMP was investigated using the paddle method with a stirring rate of 50 rpm at 37.5 °C. Briefly, the coarse CN and CN-INMP containing an equivalent CN at a dose of 2 mg was, respectively, dispersed into 100 mL of simulated lung fluid with 0.2% of SDS [17]. At the predetermined time points, 0.5 mL samples were collected and filtered using a 0.1 μm of syringe filter, and an equal volume of fresh medium was replenished immediately. The filtrate was collected for the determination of CN by HPLC with UV detector and equipped with a C18 reversed-phase column (Agilent 1200, Santa Clara, CA, USA). The mobile phase was composed of acetonitrile and 0.1% aqueous phosphoric acid solution (60:40, *v*/*v*). The UV absorption wavelength was 428 nm. The flow rate and retention time was 1 mL/min and 4.5 min, respectively. The measurements were conducted in duplicates.

#### 2.2.9. Scanning Electron Microscopy (SEM)

The particle morphology of all the coarse CN and CN-INMP were studied using a scanning electron microscope. The test powder samples were gold coated with platinum at 10 mA for 30 s using a sputter coater (Fison Instruments, Glasgow, UK). The samples were then examined using the X650 scanning electron microscope (SEM) (Hitachi, Tokyo, Japan) at 5.0 kV.

#### 2.2.10. Differential Scanning Calorimetry (DSC)

Thermograms of the CN-INMP were studied using a Diamond DSC (Perkin-Elmer, Waltham, MA, USA). The temperature was firstly calibrated using indium and tin as reference materials. The test CN-INMP samples were heated in aluminum pans under nitrogen purge from 20 °C to 260 °C at a heating rate of 10 °C/min. The measurements were conducted in duplicates.

#### 2.2.11. Powder X-ray Diffraction (PXRD)

The powder crystallinity of the coarse CN, matrix formers and CN-INMP were evaluated using D8ADVANCE X-ray diffractometer (BRUKER AXS GMBH, Karlsruhe, German) with Cu-Kα radiation at 40 mA and 45 kV. The patterns were recorded at a scan angular speed of 2 °/min and a step length as 2θ from 5° to 90°. The measurement was repeated in triplicate.

#### 2.2.12. In Vitro Aerodynamic Performance of CN-INMPs

The CN-INMPs were evaluated in vitro using a Next Generation Impactor (NGI, Beijing Huironghe Technology Co. Ltd., Beijing, China). This assay was applied as in the previously described method [27]. In each experiment, ten capsules, each filled with 20 ± 1 mg of the test formulation, were actuated. The test formulations were evaluated by the dry powder inhaler device HUARUI^®^ (BDD06, Shanghai, China) with a flow rate of 95 L/min and actuation time of 2.4 s. For each experiment, the perspirator was filled with 20 mL of the collection solvent, and stages were not coated. Particles deposited on the capsule shells, the inhalation device, the induction port, the mouthpiece adapter, the perspirator, and the impactor stages were collected by rinsing with the collection solvent. The in vitro aerodynamic performance was expressed in terms of the mass median aerodynamic diameter (AD_e_) and the fine particle fraction (FPF%). The fine particle fraction was calculated as the ratio (% *w*/*w*) of the amount of the drug in particles with an aerodynamic diameter smaller than 5.00 μm to the amount of the drug emitted from the device.

#### 2.2.13. Cell Viability and Cell Uptake

The Beas-2B cell line was obtained from KeyGEN BioTECH Co., Ltd. (Nanjing, China). The cell was cultured in a BEBM medium contain 90% RPMI-1640 and 10% FBS, and incubated at 37 °C with 5% CO_2_. The Beas-2B cells were firstly seeded in 96-well plates (1 × 10^5^ cells/mL) and cultured for 24 h before drug treatment. Then, the different concentrations of CN-NS were dropped into the cell wells. After treatment for 24 h, the cells were incubated with 10 μL of CCK8 (KeyGEN BioTECH, Nanjing, China) at 37 °C for 4 h. Then, the optical density was measured at 450 nm with a microplate plate reader (BioTek ELx800, Winooski, VT, USA).

The A549 cell line was obtained from KeyGEN BioTECH Co., Ltd. (Nanjing, China). The A549 cell was cultured in a medium contain 90% F-12K and 10% FBS. The cells were incubated at 37 °C with 5% CO_2_. The cellular uptake evaluation of CN-NSs was determined using flow cytometer. After culturing for 2 days, the A549 cells were treated with 1 mL CN-NSs (50 μg/mL) at 37 °C for 2 h. Additionally, the culture medium was removed, the cells were washed 2–3 times with PBS, trypsinized and centrifuged. The supernatant was removed, and the centrifuged cells were gently resuspended in 0.25 mL PBS. The mean fluorescence intensity of cells was determined using the flow cytometer.

#### 2.2.14. Pharmacokinetic Study in Rats of CN-INMPs

##### Drug Retention in Lung

Animal welfare and experimental procedures were performed according to the Guide for the Care and Use of Laboratory Animals and the ethics regulations of Jiangxi University of Traditional Chinese Medicine. The male rats were firstly anesthetized with an i.p. injection of ketamine/xylazine cocktail. The CN-INMP was intratracheally administered using a lavage needle at a dose of 10 mg/kg. Six rats for each time point were euthanized at predetermined time points (0.5 h and 2 h) after pulmonary administration, and the lungs were collected and washed for analysis. The obtained lungs were homogenized by adding an 80% acetonitrile solution at a ratio of 1:3 (*w*/*v*). Then, the tissue homogenates were centrifuged using high-speed freezing centrifuge at 6000 rpm for 10 min at 4 °C, and the obtained supernatant was stored at −20 °C. Each tissue sample (50 μL) was vortex-mixed with 100 μL of emodin solution (100 ng/mL) as an internal standard (IS). After centrifuging for 10 min at 12,000 rpm and 4 °C, 10 μL of supernatant sample was assayed using HPLC/MS/MS. The full scan spectra of samples showed the parent ion at m/z 367.1/134.0 for CN and m/z 269.0/225.0 for IS, respectively. An elution system of aqueous formic acid and acetonitrile at a ratio of 40:60 was used. The flow rate was set as 1 mL·min^−^^1^.

The adult male Sprague-Dawley (SD) rats (180–220 g) were divided into the following three groups: (A) CN-INMP@TPGS, (B) CN-INMP@P407, and CN-INMP@TW80. Group A, B and C were subjected to pulmonary administration by dry powder insufflators. The administration dose of each group was equivalent to 10 mg/kg of CN. At least six rats were tested for each time point (10, 15, 30, 60, 120, 240, 360, 480, 720 and 1440 min). Approximately 0.5 mL of blood was withdrawn from the orbital plexus at predetermined time intervals post administration. The blood samples were centrifugated at 6000 rpm for 10 min (4 °C), then the plasma was obtained and stored at −20 °C until further analysis. The CN plasma concentrations were assayed by a validated HPLC/MS/MS method as described above.

##### Statistical Analysis

Next, DAS 2.1.1 was used for the pharmacokinetic parameters analysis. Two-way or three-way analysis of variance (ANOVA) were used for statistical comparison. The significance level was set to 0.05 unless otherwise stated.

## 3. Results and Discussion

### 3.1. The Particle Size and Morphology of CN-NS

**Figure 2:**
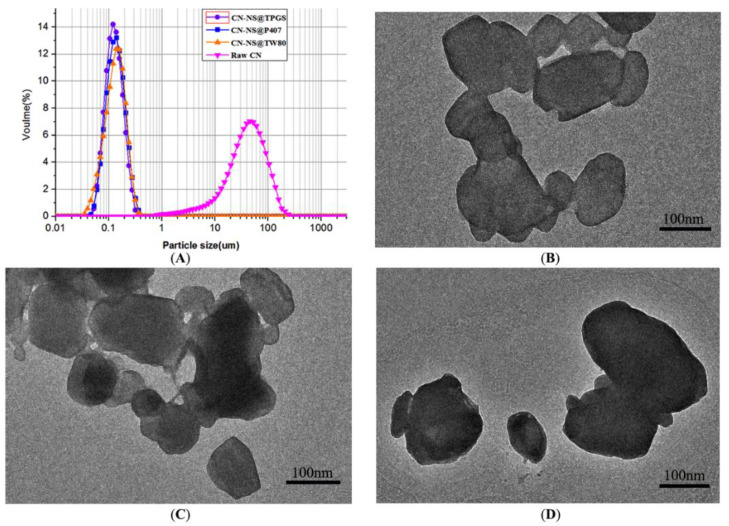
The particle sizes (**A**) of the CN-NS modified by different stabilizers, morphology of CN-NS@TPGS (**B**), CN-NS@TW80 (**C**), CN-NS@P407 (**D**).

Stabilizer was an important factor influencing particles size and the zeta potential of drug nanocrystals [28]. The particle sizes of the CN-NS modified by different stabilizers are shown in Figure 2A. The results showed that the volume mean particle sizes(D50) of the freshly prepared CN-NS@TPGS, CN-NS@P407, CN-NS@TW80 were 119.1 ± 2.12 nm, 121.87 ± 1.31 nm, and 131.08 ± 5.78 nm, respectively. However, the D50 of raw CN was 56.29 ± 1.85 µm. Additionally, there was no significant difference among the particles size of different CN-NSs. The morphology images of CN-NS@TPGS, CN-NS@P407, and CN-NS@TW80 are showed in Figure 2. The CN-NS seemed to be irregular shaped particles with a particle size of 120 nm (Figure 2B–D). The Zeta potential of CN-NS@TPGS, CN-NS@P407, and CN-NS@TW80 was −26.5 ± 0.3 mV, −14.7 ± 1.08 mV, and −3.2 ± 0.6 mV, respectively. It could be concluded that the CN-NS@TPGS exhibited a relatively lower negative charge potential compared with CN-NS@P407 and CN-NS@TW80. However, all the CN-NSs seemed to be smaller than mucus pore sizes in order to permeate through them [29,30].

### 3.2. The Mucointeraction between Mucin and CN-NS

**Figure 3:**
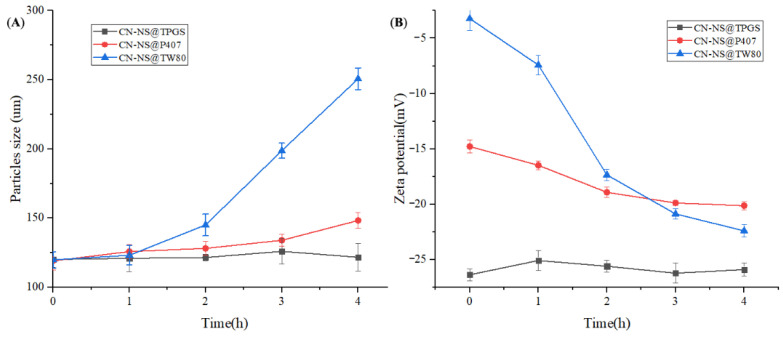
The changes in Zeta potential (**A**) and particle size (**B**) of the CN-NS incubated with mucin.

The change in particle size of the CN-NS incubated with mucin is shown in Figure 3. Figure 3A displays that the particle size of CN-NS@P407 and CN-NS@TW80 remarkably increased, but those of CN-NS@TPGS only demonstrate slight variation. The zeta potential results of CN-NS@TW80 and CN-NS@TPGS are shown in Figure 3B. The Zeta potential of CN-NS@TW80 remarkably decreased with interaction time, but the variation of the Zeta potential of CN-NS@TPGS seemed not to be obvious. These indicated that the CN-NS@TW80 could form a large complex with the mucin, owing to the interaction effect. However, the CN-NS@P407 and CN-NS@TPGS had a slight interaction with mucin. It could be due to the fact that the mucin was a highly negatively charged hydrophilic linear peptide chain consisting of 8–169 amino acids of repeated proline, threonine, and serine domain [31,32]. The lung mucus was prone to trapping and removed nanoparticles by a variety of adhesive interactions such as hydrogen-bonding, electrostatic and hydrophobic interactions. The nanoparticles with a strong negative potential and hydrophilic surface could avoid the interaction effect in order to penetrate the mucus layer [33]. As displayed in Figure 3B, the CN-NS@TPGS exhibited a much lower Zeta potential and more hydrophilic potential in comparison to the CN-NS@P407 and CN-NS@TW80. TPGS modification could provide a hydrophilic protective layer and negatively charged surface potential which could repel the adsorption of mucin proteins via the steric barrier and strong electrostatic repulsion [34,35]. Therefore, the CN-NS@TPGS would experience little interaction with the negatively charged mucin due to the strong electrostatic repulsion effect and long PEG chain.

### 3.3. The Mucus Penetration Ability of CN-NC in Mucus

**Figure 4:**
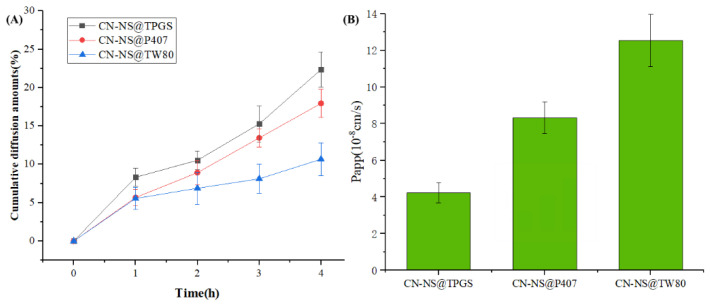
The cumulative diffusion amount (%) (**A**) and permeability coefficient (**B**) of the CN-NS incubated with mucin.

The ability of different CN-NSs to penetrate through the mucus layer was quantified using a transwell system. Figure 4A shows the cumulative penetrating ability of different CN-NS in the mucus. Figure 4B displays the *P_app_* values of different CN-NS in the mucus. The penetrating ability and permeability coefficient (*P_app_* = 12.53) of the CN-NS@TPGS in mucus were significantly enhanced compared with the CN-NS@P407 (*P_app_* = 8.32) and CN-NS@TW80 (*P_app_* = 4.24). These further proved that TPGS-modified CN-NS exhibited a much higher penetration ability across the mucus layer compared with CN-NS@P407 and CN-NS@TW80, which could effectively block the adhesive interactions with mucus components.

### 3.4. The Mucus Penetration Evaluation of CN-NS in Mucus by CLSM

**Figure 5:**
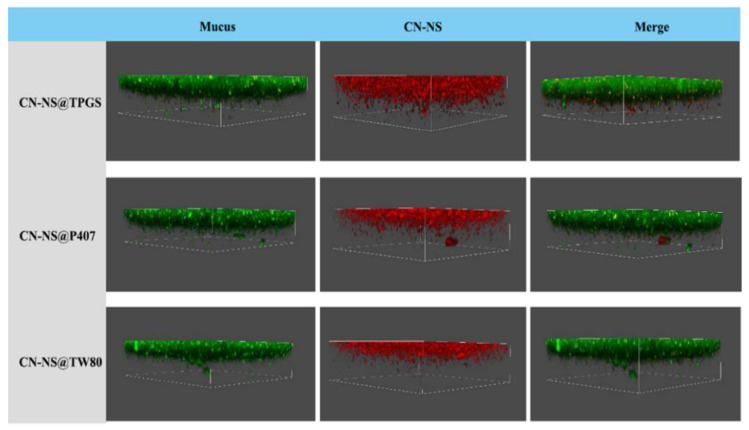
Three-dimensional imaging of the distribution of CN-NSs in mucus.

To directly observe the distribution of CN-NS in the mucus layer, the diffusion behavior of CN-NS was evaluated using confocal laser scanning microscopy (CLSM). Figure 5 illustrates the 3D images of CN-NS@TPGS, CN-NS@TW80 and CN-NS@P407 in mucus, and it was found that the CN-NS@TPGS mostly presented in deep layers of mucus along the z-direction, while the CN-NS@P407 and CN-NS@TW80 were mainly localized in the upper layer of mucus. These indicated that CN-NS@TPGS could more easily penetrate through the mucus layer, in comparison with CN-NS@P407 and CN-NS@TW80.

### 3.5. The Aerodynamic Performance and Redispersibility Evaluation of CN-INMP

**Figure 6:**
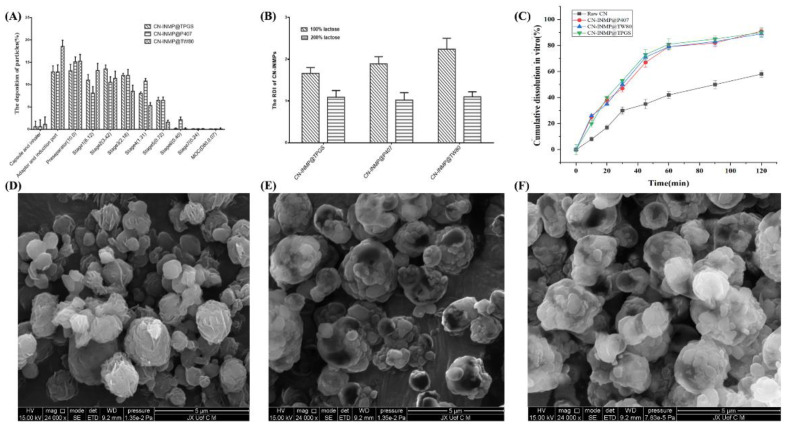
The lung deposition property (**A**), redispersibility index (**B**), the cumulative dissolution (**C**) and morphology of CN-INMP@TPGS (**D**), CN-INMP@P407 (**E**), and CN-INMP@TW80 (**F**).

The mass median aerodynamic diameter (AD_e_) and the fine particle fraction (FPF%) can generally be used as screening parameters to assess the aerodynamic performance and lung deposition ability of inhalable CN-INMPs. All the tested CN-INMPs had a relatively low tap density in the range of 0.302–0.431 g/mL (shown in Table 1). As displayed in Table 1 and Figure 6A, the AD_e_ of all of the investigated CN-INMPs were 3.722–4.582 μm. It can be seen that the AD_e_ of the CN-INMPs obtained by NGI seemed suitable for the aerodynamic requirement of inhalation administration [36].

The results also showed that the concentration of lactose as matrix formers remarkably influenced the AD_e_ value of tested CN-INMPs (*p* < 0.05), and the FPF value of tested CN-INMP increased remarkably with the increase in the lactose concentration (*p* < 0.05). The results also showed that the FPF of CN-INMP@P407 was not remarkably different to those of CN-INMP@TPGS and CN-INMP@TW80 using 200% of the lactose as matrix formers. This could be attributed to the suitable deposition efficiency of CN-INMP@P407 owing to a decreased adhesion among particles. However, using 100% of LA as the matrix former, the CN-INMP@TPGS and CN-INMP@TW80 exhibited similar ADe, but lower FPF values were obtained, which could be attributed to strong adhesion among particles and poor flowability owing to their hygroscopicity. Therefore, the 200% concentration of LA as matrix formers seemed to be the optimum choice for CN-INMPs with excellent lung-deposition performance.

The redispersibility index (RDI) could be used to evaluate the reconstitution ability of CN-INMP, indicating whether the CN-INMP could completely redisperse into CN-NS in the simulated lung fluid. The RDI of tested CN-INMPs were displayed in Figure 6B. It could be observed that the RDIs of the CN-INMP@TPGS, CN-INMP@P407, CN-INMP@TW80 with 100% lactose were obviously higher than those of CN-INMPs with 200% lactose. The RDIs of CN-INMP with 200% lactose were near to 1, which indicated that the CN-INMPs could completely reconstitute the original CN-NS. However, 100% lactose as matrix formers could not effectively prevent the aggregation of CN-INMP@TPGS and CN-INMP@TW80 (RDI > 1.5). This might be related to a low glass-transition temperature of TPGS and TW80, thus could not adequately prevent CN-NS from aggregations during spray-drying. The dissolution behaviors of CU-INMPs with 200% lactose was investigated in simulated lung fluid. As displayed in Figure 6C, approximately 47% CN dissolved from the CN-INMP@TPGS, CN-INMP@P407, CN-INMP@TW80 within 30 min, respectively. After 120 min, approximately 89% CN dissolved from the three CN-INMP groups. For the coarse CN group, only 30% CN was dissolved within 60 min, and 58% was dissolved after 2 h. This demonstrated that all the CN-INMPs exhibited faster dissolution in comparison to the coarse CN, owing to the particles size reduction of drug [9,10]. The morphology images of CN-INMPs are displayed in Figure 6D–F. All the CN-INMP@TPGS, CN-INMP@P407, CN-INMP@TW80 seemed to be sphere particles. Additionally, the structure of CN-NS agglomerates in CN-INMPs could also be found on the surface of particles, which indicated that the CN nanocrystals were successfully embedded into the matrix former [13,26].

### 3.6. Crystallinity of Spray-Dried CN-INMPs

**Figure 7:**
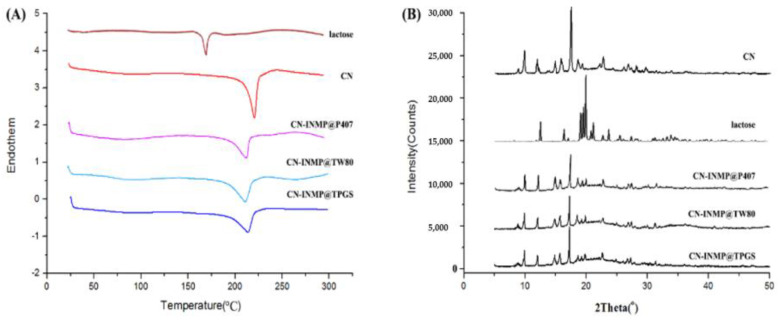
The DSC (**A**) and XRD (**B**) diffractograms of coarse CN, lactose, CN-INMP@TPGS, CN-INMP@P407 and CN-INMP@TW80.

DSC thermograms of coarse CN, LA, CN-INMP@TPGS, CN-INMP@P407 and CN-INMP@TW80 are presented in Figure 7A. The coarse CN had a melting peak at 182 °C. LA exhibited a melting peak at 165 °C. For the spray-dried CN-INMP@TPGS, CN-INMP@P407 and CN-INMP@TW80, the melting peaks of CN were shifted to 178 °C, but its enthalpy was decreased owing to the decreased particle size. However, the melting peaks of LA in CN-INMP seemed to be absent, which meant that LA could be the amorphous state in CN-INMP. Figure 7B displays the XRD diffractograms of coarse CN, lactose, CN-INMP@TPGS, CN-INMP@P407 and CN-INMP@TW80. The coarse CN exhibited characteristic crystalline peaks at 2θ of 10.2 and 17.1°. The characteristic peaks of the matrix former LA were seen at 2θ of 19.9° and 20.9°. The characteristic peaks of CN at of 10.2 and 17.1° were present in the spray-dried CN-INMP@TPGS, CN-INMP@P407 and CN-INMP@TW80, which indicated that the presence of CN in the crystalline state. However, the amorphous halo of lactose seemed to be not obviously observed in CN-INMPs, which could be dye to the characteristic peaks of CN in the spray-dried CN-INMPs strongly “masking” the amorphous halo peak of lactose at 2θ of 20° [37]. It was found that after spray-drying, the crystalline state of CN might be constrained owing to glassy matrix of amorphous LA. However, the amorphous matrix-former of CN-INMP was likely to transform from a glass state to rubbery state at a high moisture content and storage temperature conditions [38]. Therefore, the CN nanocrystals embedded into the amorphous matrix also might generate irreversible aggregation during storage.

### 3.7. Cell Viability and Cell Toxicity

**Figure 8:**
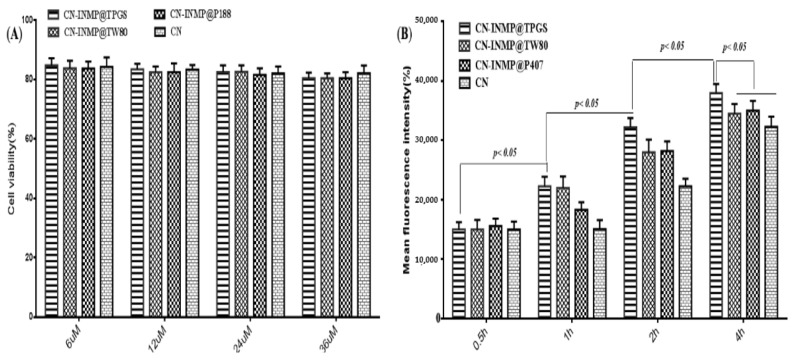
The cell viability(**A**) and cell uptake(**B**) of CN-INMPs.

In this study, the Beas-2B cell line was used as the model cell to assess the viabilities of CN-INMP. The viability of Beas-2B cells treated with different CN-INMPs was evaluated using Cell Counting Kit-8 methods. The results show that the viabilities for all the CN-INMPs did not change remarkably with the increasing concentration (*p* > 0.05), as shown in Figure 8A. The viabilities of CN-INMPs were all higher than 80%, suggesting that all the CN-INMPs showed no cellular toxicity from a 6 to 36 µM concentrations. To ensure the viability of cells during uptake, the 24 µM concentration was chosen for further cell study. The cellular uptake of CN-INMP was quantitatively assayed by flow cytometry. The results displayed that the mean fluorescence intensity of the CN-INMP@TPGS group after incubation for 4 h was significantly higher in comparisons with that of CN-INMP@P188 and CN-INMP@TW80 (*p* < 0.05), respectively. Additionally, as shown in Figure 8B, the cellular uptake levels of all of the tested groups indicated a time-dependent relationship (*p* < 0.05).

### 3.8. In Vivo Pharmacokinetic Study of CN-INMPs

**Figure 9:**
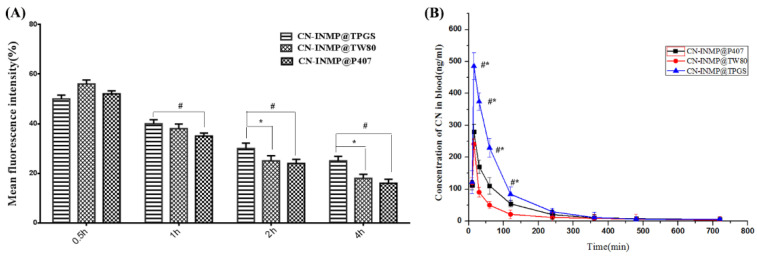
(**A**) the retention of CN in lung after pulmonary administration of tested CN-INMPs from 0.5 h to 2 h, and (**B**) the concentration of CN in blood after pulmonary administration of tested CN-INMPs. # The significance (*p* < 0.05) between CN-INMP@TPGS and CN-INMP@P407 formulation. * The significance (*p* < 0.05) between tested CN-INMP@TPGS and CN-INMP@TW80.

The amount of CN-INMP retained in the lung at different time intervals was then determined quantitatively. Figure 9A shows that the retention of CN-NS@TPGS in lung was not significantly different to that of CN-INMP@TW80 groups (*p* < 0.05) at 0.5 h post administration. Additionally, the drug concentration in blood for the CN-INMP@TPGS group was also significantly higher than that of the CN-INMP@P407 (*p* < 0.05). This could be attributed to the fast dissolution behavior of CN-INMP and the obstacles presented by the mucus. However, 2 h post administration, the retention of CN in lung was further decreased in tested groups. The drug retention amount of the CN-INMP@TPGS group was still higher than for the other two groups (*p* < 0.05), while the difference between CN-INMP@TW80 and CN-INMP@P407 was not obvious. It was concluded that the difference in the retention among CN-INMP@TPGS, CN-INMP@TW80 and CN-INMP@P407 could be attributed to the surface modification of nanocrystals.

The plasma concentration profiles of CN-INMP@TPGS, CN-INMP@TW80 and CN-INMP@P407 after the pulmonary administration of CN-INMP are presented in Figure 9B, and the main pharmacokinetics parameters were calculated and are listed in Table 2. It could be observed that the plasma concentrations of the inhalable CN-NS@TPGS were significantly higher compared to those of the CN-INMP@TW80 and CN-INMP@P407 (*p* < 0.05) within 2 h following pulmonary administration. Among the three groups, the C_max_ (485.23 ± 18.11 µg/L) of the CN-INMP@TPGS group significantly increased (*p* < 0.05), compared with those of CN-INMP@TW80 (289.26 ± 12.38 µg/L) and CN-INMP@P407 (242.42 ± 29.59 µg/L). The AUC_(0-t)_ value of CN-INMP@TPGS (2413.18 ± 432.41 µg/L h) was 1.497- and 3.32-fold greater compared with those of CN-INMP@TW80 (1612.35 ± 261.35 µg/L h) and CN-INMP@P407 (777.59 ± 196.81 µg/L h), respectively. These results indicated that the CN-INMP@TPGS was absorbed rapidly after pulmonary administration and resulted in increased systemic absorption. This might be attributed to the excellent redispersibility of CN-INMP@TPGS after lung deposition. Additionally, TPGS modified CN-NS after redispersion exhibited an excellent penetrating capability in lung mucus, owing to the negative potential surface and PEGylating modification [39]. Consequently, the inhalable CN-INMP could improve the in vivo bioavailability of CN after pulmonary administration remarkably.

## 4. Conclusions

In the present study, we fabricated three surface-modified inhalable nanocrystal-in-microparticles for pulmonary delivery and studied the effect of different types of surface modification on the mucus penetration in vitro and absorption in vivo. The results demonstrated that TPGS- modified nanocrystals could improve the retention time in lung, plasma concentrations and the systemic absorption of CN after pulmonary administration. The improved absorption of CN-NS@TPGS was attributed to the increase in the mucus-penetrating ability and cellular uptake in lung epithelium cells. Additionally, CN-INMP could also enhance the lung deposition of CN after pulmonary administration. Therefore, the mucus-penetrating nanocrystals-in-microparticles might be used as a promising formulation strategy for the inhalation administration of poorly soluble drugs.

## Figures and Tables

**Table 1:** The particle size and aerodynamic parameters of CN-INMPs ($\bar{x}$ ± s, *n* = 3).

Stabilizer	Formers/Drug Ratio (%)	Carr Index (CI)	ρ	AD_e_ (μm)	FPF%
TPGS	100%	29.13	0.352	4.537 ± 0.050	31.32 ± 0.020
	200%	27.41	0.392	3.924 ± 0.010	46.737 ± 0.020
P407	100%	27.52	0.412	4.210 ± 0.123	42.31 ± 0.020
	200%	24.35	0.431	3.722 ± 0.020	51.25 ± 0.010
TW80	100%	34.67	0.302	4.582 ± 0.132	25.32 ± 0.110
	200%	31.12	0.341	4.221 ± 0.451	31.302 ± 0.300

**Table 2:** The main pharmacokinetic parameters of the tested CN-INMPs after pulmonary administration at a dose of 20 mg/kg ($\bar{x}$ ± s, *n* = 6).

**Formulation**	**C_max_ (µg/L)**	**T_max_ (h)**	**MRT_(0-t)_ (h)**	**AUC_(0-∞)_ (µg/L h)**
CN-INMP@TPGS	485.23 ± 18.11 ^a,b^	3.37 ± 1.37 ^a,b^	4.24 ± 1.14 ^a,b^	2413.18 ± 432.41 ^a,b^
CN-INMP@P407	289.26 ± 15.38 ^c^	2.12 ± 1.09	2.67 ± 0.42 ^c^	1612.35 ± 261.35 ^c^
CN-INMP@TW80	242.42 ± 13.59	2.16 ± 1.08	1.75 ± 0.35	777.59 ± 196.81

^a^ The significance (*p* < 0.05) between CN-INMP@TPGS and CN-INMP@P407. ^b^ The significance (*p* < 0.05) between CN-INMP@TPGS and CN-INMP@TW80. ^c^ The significance (*p* < 0.05) between CN-INMP@P407 and CN-INMP@TW80.

## Data Availability

Data is contained within article.
